# Treatment of United Fractures

**Published:** 1897-03

**Authors:** William Perrin Nicolson

**Affiliations:** 78 Marietta Street; Atlanta, Ga.


### Southern Medical Record.

A MONTHLY JOURNAL OF MEDICINE AND SURGERY.

Vol. XXVII. ATLANTA, GA., MARCH, 1897. No. 3

### Original Articles.

### TREATMENT OF UNUNITED FRACTURES.

By WILLIAM PERRIN NICOLSON, M. D., Atlanta, Ga.

Operations for ununited fractures by wiring the fragments are sufficiently infrequent and the results of enough uncertainty to justify a report of such cases. My experience has been limited to three operations, the results of which have differed materially, though apparently equal care was exercised in the attempt to secure union.

Case I.—James S., white, aged 52, received a fracture of the right forearm, caused by being thrown under a street car. The fracture was complicated by somewhat severe contusion of the soft parts. Dressings were applied by physicians in the neighborhood, and when I saw him three days later there was great swelling about the seat of the fracture, the wire being covered with large blebs. A perforated metallic splint was adjusted, and wet antiseptic dressings applied for some days. The fracture apparently healed kindly, and at the end of five weeks the splint was removed and the bones were apparently firmly united, with a good result. One week later the patient informed me that he could detect uration at the point of fracture, by fixing the palm of the hand upon a table and alternately contracting the flexion and exterior muscles of the forearm. At the end of another week, this was well marked and the limb was again put in a fixed dressing and so maintained for several weeks. But in spite of all treatment the union was gradually absorbed until the bones were held only by fibrous tissue, which permitted free motion at the seat of fracture. As the patient was entirely unable to pursue his occupation, he consented to an operation. At the Surgical Clinic of the Southern Medical College, I operated, making an incision four inches long on the inner margin of the radius. An oblique fracture was found, united by fibrous tissue. This was cut away with the aid of gouge and chisel, and the fragments drilled and brought together with short silver wire. The ulna was similarly treated, only the fragments were left in opposition without wiring. Over the gauze dressing a plaster of Paris bandage was applied. This remained in position only four days, when it was removed on account of pain and swelling which proved to result from suppuration of the radial wound. The forearm was bandaged to an anterior splint and antiseptic dressings applied to the suppurating wound. At last dressing, it could be clearly seen that union was more solid, and in the course of four weeks, the wounds were healed and the union in the radius was complete. The ulna did not attain to bony union.

In the course of a few weeks, the patient resumed his occupation, carrying heavy weights, and at this time, eight years afterward, the extremity is as strong as ever.

CASE II.—L. H., white, aged 30, motorman on trolley-car, in collision with another car, was caught between the dashboards and sustained fracture of both thighs. He was admitted to the private ward of the Grady Hospital and the fracture was dressed by the surgeon of the railroad company. At the end of one week he was transferred to the charity ward and came under the service of Dr. H. P. Cooper, who recognized a lapping of the bones and attempted by traction under anesthesia to overcome the deformity. Failing in this, both limbs were put up in plaster of Paris dressing which was used continuously for about two months. At the end of this time union in the right femur was fairly firm, but in the left there was practically non-union. The fragments could be mapped out, the lower projecting almost under the skin in the popliteal space, the upper extending just above patella.

Under ether, an incision was made along the outer aspect of the thigh seven inches over the external inter-muscular septum; working down to the fragments, the lower was easily reached, lying not much below the surface in the popliteal space.

It presented a transverse line of fracture and was devoid of an appearance of callus. The upper fragment was surrounded by a mass of callus as large as an orange, which involved the surrounding muscles very closely. This was gouged and chiseled out as far as possible by a most tedious dissection, the immediate proximity of the femoral vessels to its inner and unexposed side, rendering this a somewhat perilous proceeding. The fragment was finally sufficiently released to permit of passing the chain saw and removing about two inches of the end of the bone. The lower fragment was similarly treated and both upper and lower ends drilled. It was found that on account of the displacement of the upper fragment that the ends could not be brought into direct opposition, but only about one-third of the diameter could be brought together. A strong silver wire was passed and twisted as far as possible and the cut ends pushed between the bones. The wound was closed with catgut and a catgut drain brought out at the lower angle. A plaster of Paris dressing was applied from the toes to the waist. Time of operation and dressing two hours and fifty minutes. At the end of the first week a window was cut over the wound and new dressings applied. There was no symptom of any moment during the convalescence. Highest temperature was 101° on the third day. Plaster dressings were continued six weeks and then short splints to thigh two weeks more. Union was solid and perfect, and two years afterward the patient has no trouble except that he is four inches shorter than before the injury.

CASE III.—D. H. O., physician, aged 24, while driving to see a patient one year before, a cyclone passed across his track, blowing a tree upon his buggy and fracturing both thighs His injuries were so serious that the attending physician thought he could not recover, and little was done for his broken bones. When it was found that he would recover, the bones were lapped beyond hope of replacement. When I saw him a year later, he walked with difficulty on crutches, there being but poor union in the right femur and none at all in the left. The site of fracture in the left was at the junction of the upper and middle thisos. He readily consented to an operation, which was performed in April, 1894. An incision similar to that in case two, revealed the fragments lapped, the upper pointing downward and inward, and the lower upward and outward. The end of the lower was easily dislodged and cut off, but the upper being drawn under the femoral vessels was released with much difficulty. Both fragments were practically free from any deposit of callus, but the ends were rounded off and covered with fibrous tissue, differing from the lower fragment in case two, which presented a fractured surface unchanged in any way. Both fragments being cut off and drilled, not much difficulty was experienced in holding them together, and plaster dressing was applied as in the preceding case. The subsequent history was about the same. The plaster dressing remained until the fourth week when I was much disappointed to find that there was practically no union. Three weeks more of fixation showed improvement and increased strength of the bone over the condition before operation, but there was not much bony union.

In reviewing these cases, several facts are brought out. In the first, we have an example of the wrong course in a fracture, in spite of careful work and apparently good result. The abruption of an established union has been recorded in a number of instances. This is the second instance in which I have observed it, the other occurring in the humerus. Just what factors are responsible for it is an uncertain question.*

The second case is an illustration of a difficulty encountered in double fracture of the femur, which I have not seen mentioned, and that is the care that should be taken to ascertain positively that the fragments are in position. Where only one femur is fractured we have the sound limb for a guide as to length, but in the double fractures this can not be utilized. In both the second and third cases, there was lapping amounting to about four inches, resulting not only in poor union but in leaving the patient that much shorter than before the injury. The effect of this is bad in disturbing the proper proportion between the trunk and lower extremities.

The operation of wiring the femur, on account of the large amount of tissue surrounding the bone, necessitates a very large wound and altogether makes a very difficult operation as compared with more exposed wounds. It can certainly be classed as a major operation, though in the prolonged work in the two cases recorded, the patients stood it very well. It is undoubtedly a perfectly justifiable procedure in ununited fracture.

*78 Marietta Street.*

*This case also bears out a statement made by Dr. Ashurt, in a recent article, in which he claims that the present methods of surgery in compound fractures, while greatly lessening mortality by the prevention of suppuration, does give in many instances delayed union. Here we have the bone in which suppuration occurred, giving firm union, and non-union in that in which it was prevented.

